# Gefitinib loaded folate decorated bovine serum albumin conjugated carboxymethyl-beta-cyclodextrin nanoparticles enhance drug delivery and attenuate autophagy in folate receptor-positive cancer cells

**DOI:** 10.1186/s12951-014-0043-7

**Published:** 2014-10-30

**Authors:** Yijie Shi, Chang Su, Wenyu Cui, Hongdan Li, Liwei Liu, Bo Feng, Ming Liu, Rongjian Su, Liang Zhao

**Affiliations:** College of Pharmacy, Liaoning Medical University, Jinzhou, 121000 P R China; College of Veterinary Medicine, Liaoning Medical University, Jinzhou, 121000 P R China; National Vaccine & Serum Institute, Beijing, 100024 China; Central Laboratory of Liaoning Medical University, Jinzhou, 121000 P R China

**Keywords:** Folate, Folate receptors, Carboxymethyl-β-Cyclodextrin, Bovine serum albumin, Nanoparticles, Gefitinib

## Abstract

**Background:**

Active targeting endocytosis mediated by the specific interaction between folic acid and its receptor has been a hotspot in biological therapy of many human cancers. Various studies have demonstrated that folate and its conjugates could facilitate the chemotherapeutic drug delivery into folate receptor (FR)-positive tumor cells *in vitro* and *in vivo*. In order to utilize FA-FR binding specificity to achieve targeted delivery of drugs into tumor cells, we prepared Gefitinib loaded folate decorated bovine serum albumin conjugated carboxymethyl-β-cyclodextrin nanoparticles for enhancing drug delivery in cancer cells. On this context, the aim of our study was to develop a novel nano-delivery system for promoting tumor-targeting drug delivery in folate receptor-positive Hela cells.

**Results:**

We prepared folic acid (FA)-decorated bovine serum albumin (BSA) conjugated carboxymethyl-β-cyclodextrin (CM-β-CD) nanoparticles (FA-BSA-CM-β-CD NPs) capable of entrapping a hydrophobic Gefitinib. It was observed that nanoparticles are monodisperse and spherical nanospheres with an average diameter of 90.2 nm and negative surface charge of −18.6 mV. FA-BSA-CM-β-CD NPs could greatly facilitate Gefitinib uptake and enhance the toxicity to folate receptor-positive Hela cells. Under the reaction between FA and FR, Gefitinib loaded FA-BSA-CM-β-CD NPs induced apoptosis of Hela cells through elevating the expression of caspase-3 and inhibited autophagy through decreasing the expressing of LC3. It also confirmed that clathrin-mediated endocytosis and macropinocytosis exerted great influence on the internalization of both NPs.

**Conclusions:**

These results demonstrated that FA may be an effective targeting molecule and FA-BSA-CM-β-CD NPs provided a new strategy for the treatment of human cancer cells which over-expressed folate receptors.

## Background

Nanosized drug carriers functionalized with moieties specifically targeting tumor cells are promising tools in cancer therapy, due to their ability to circulate in the bloodstream for longer periods and their selectivity for tumor cells, enabling the sparing of healthy tissues [1–5]. Many synthetic biomimetic nanocrystalline apatites are used as nanocarriers to produce multifunctional nanoparticles, by coupling them with the chemotherapeutic drug, such as Gefitinib, Dox or membrane antibody DO-24 monoclonal antibody (mAb) directed against the c-Met/Hepatocyte Growth Factor Receptor (Met/HGFR), which is over-expressed on different kinds of carcinomas and thus represent a useful tumor target recently [6–8]. Gefitinib, a tyrosine kinase inhibitor of Epithelial Growth Factor Receptor (EGFR) usually expressed in solid tumors of epithelial origin, can prevent tumor growth, metastasis and angiogenesis, and promote apoptosis of tumor cells [9–11]. The main mechanism includes that it can block the signal transmission by competitive binding Mg-ATP situated on catalytic domain of EGFR-TK, then inhibit the activation of mitogen activated protein kinase, inducing the apoptosis of cancer cells [12]. However, Gefitinib is absorbed slowly and widely distributed in bodies following oral administration, resulting in the serious side effects and lower bioavailability. Moreover, the solubility of Gefitinib is decreased with the decline of pH in medium [13].

Cyclodextrins (CD), a family of carbohydrate polymers which are produced from starch by enzymatic conversion and commonly used in food, pharmaceutical, drug delivery, and chemical industries, as well as agriculture and environmental engineering, is cyclic oligosaccharide with cone barrel structure composed of seven glucopyranose units with cylindrical cavity [14–16]. The exterior of this cone is polar and hydrophilic, whereas the interior cavity is relatively nonpolar and hydrophobic. Small hydrophobic molecules as the guest molecules can be completely or partially embedded into CD cavity to form complexes, improving water solubility, stability and biological activity of the guest molecules [17–21]. Bovine serum albumin (BSA), a carrier protein, plays an important role in drug storage and transport, for its superior biocompatibility it has been widely used in biomedical research, such as Nano carrier, nanoparticle surface engineering and temples for preparation of nanoparticles [22–25].

**Figure 1:**
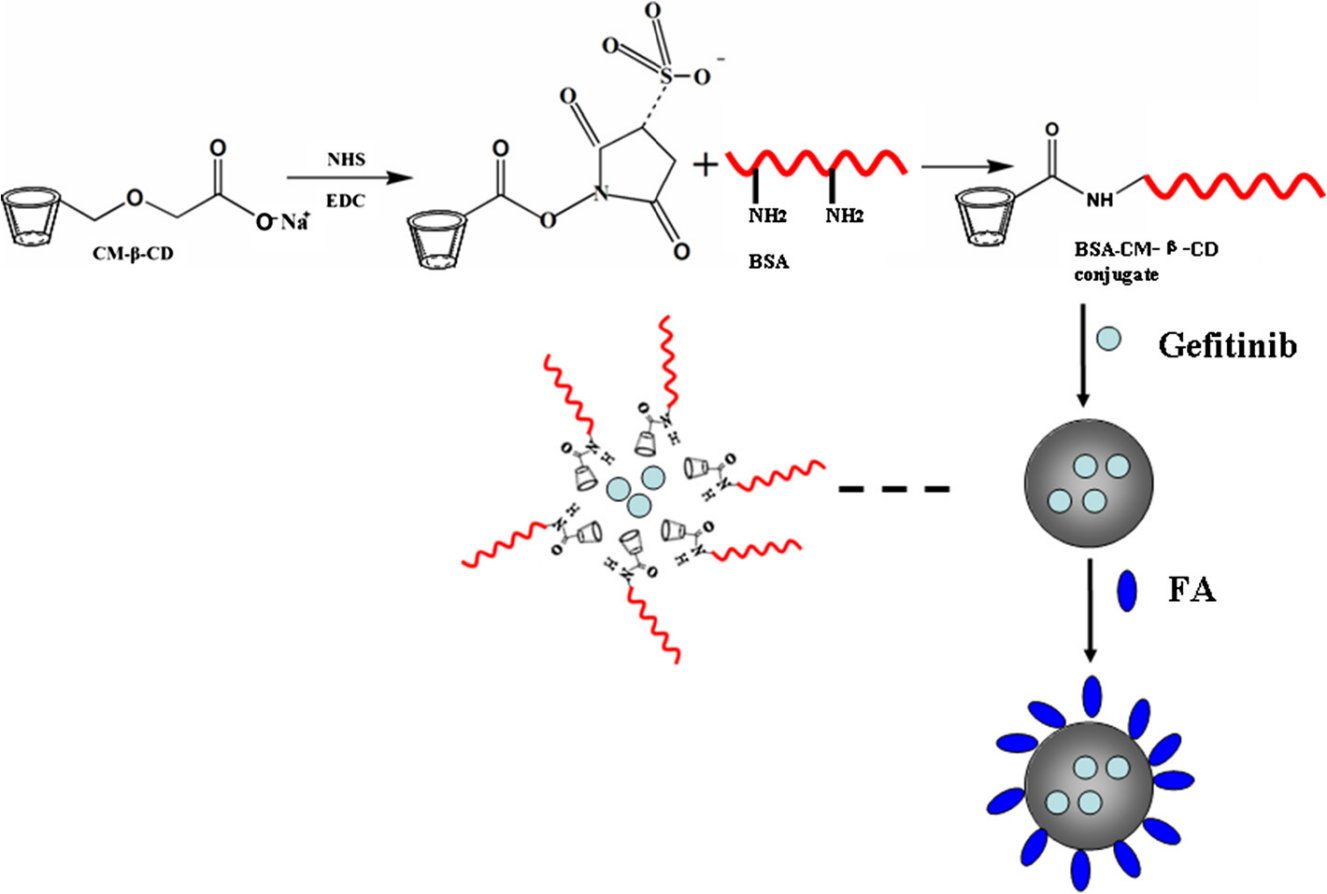
**Schematic formation of Gefitinib loaded folate-decorated bovine serum albumin conjugated carboxymethyl-β-cyclodextrin nanoparticles.**

To improve the solubility and stability of Gefitinib, we synthesize the amphiphilic BSA-CM-β-CD conjugates to prepare the assembled nanoparticles capable of entrapping hydrophobic Gefitinib into the cavity of CD through the host-guest interaction. Folate receptor (FR), as a trans-membrane glycoprotein, promotes the transportation of folate (FA) or its conjugates into the cells by active targeting endocytosis mediated through FA-FR interaction [26–28]. FA is expressed at basal levels in normal adult organs such as brain, lung and liver, but it is over-expressed in many human cancers including ovarian cancer, breast cancer, endometrial cancer, lung cancer, kidney cancer, colon cancer and nasopharyngeal carcinoma cells [29–31]. Several lines of evidence have demonstrated that FA and its conjugates could significantly enhance the drug delivery efficiency into FR-positive tumor cells both *in vitro* and *in vivo* [32–34]. Herein, FA is adopted as the coupling molecule to improve FR-positive tumor-targeted drug delivery (Figure 1). Properties of NPs such as size, morphology and surface potential were examined. Using Gefitinib as the model drug, we prepared drug-loaded nanoparticles. We found that FA-BSA-CM-β-CD NPs greatly facilitated Gefitinib uptake and enhanced the toxic effect in folate receptor-positive Hela cells. Our results demonstrated that FA-BSA-CM-β-CD NPs might be a higher efficiency drug delivery system than the conventional delivery system for the targeting therapy of FR positive human cancers.

## Results and discussion

### The preparation and characteristics of various kinds of NPs

Conjugation of CM-β-CD to BSA was obtained by carbodiimide coupling. Carboxylic group of CM-β-CD reacted with EDAC to form unstable reactive ester. With addition of NHS, semi-stable amine-reactive NHS-ester was synthesized and then mixed with BSA which containing amino group to obtain CM-β-CD conjugated BSA by stable amide bond [35,36].

**Figure 2:**
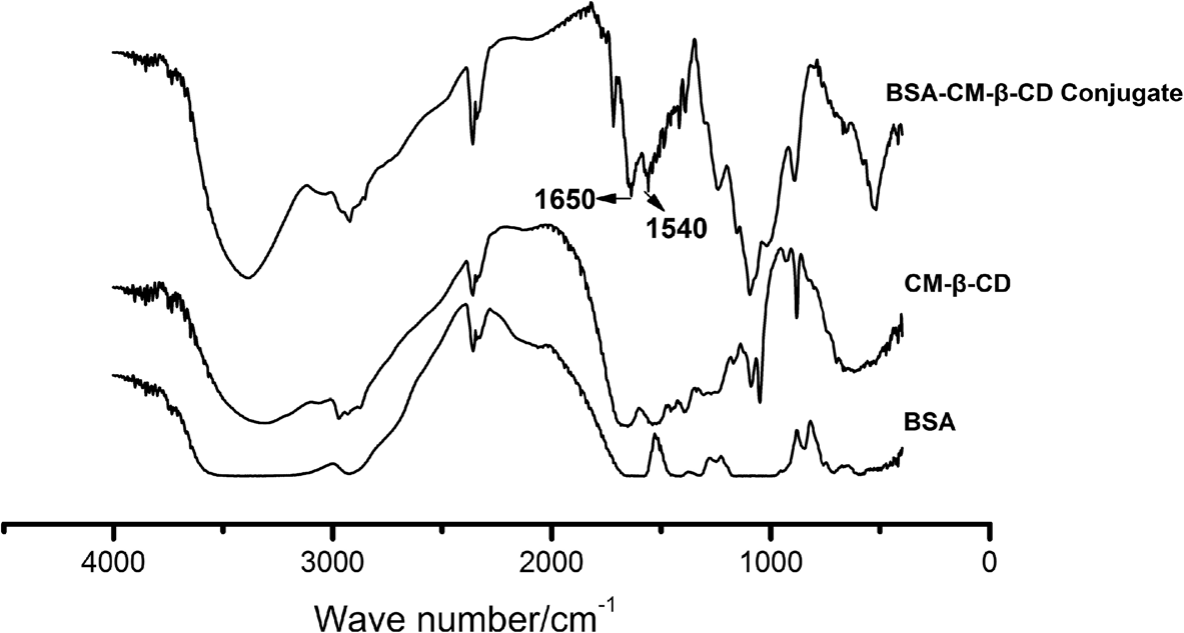
**FT-IR spectra of BSA, CM-β-CD, and BSA-CM-β-CD conjugates.**

The characterization of BSA-CM-β-CD conjugates was investigated by infrared spectroscopy (Figure 2). The result showed the FT-IR spectra of BSA, CM-β-CD, and BSA-CM-β-CD conjugates. The characteristic peak of BSA-CM-β-CD conjugates appeared at 1650 cm^−1^ and 1540 cm^−1^ should be ascribed to the newly formed amide bond between CM-β-CD molecules and BSA. These data supported that CM-β-CD has grafted to BSA, and they are correspond to the results of former literatures [37].

**Figure 3:**
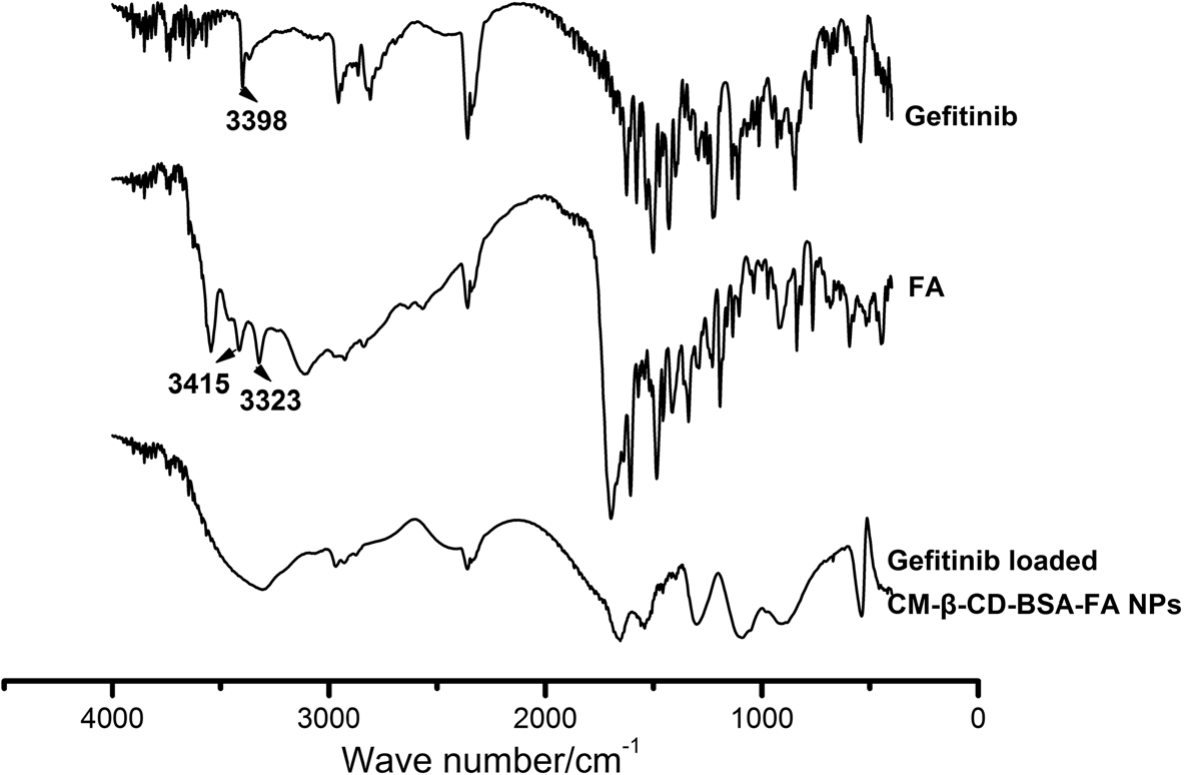
**FT-IR spectra of FA, Gefitinib and Gefitinib loaded FA-BSA-CM-β-CD NPs.**

Spectrum of infrared absorption of Gefitinib loaded FA-BSA-CM-β-CD NPs was shown in Figure 3. It can be seen that when BSA-CM-β-CD NPs was bonded with FA, its spectrum demonstrated that the aromatic amine groups (νNH_2_, 3415 cm^−1^ and 3323 cm^−1^) from FA disappeared, suggesting that amine groups from FA reacted with carboxylic group of BSA. Furthermore, the characteristic peak of secondary amine group (νNH, 3398 cm^−1^) from Gefitinib disappeared because of the encapsulation of Gefitinib into the core of NPs.

### Development and properties of Gefitinib-loaded FA-BSA-CM-β-CD NPs

**Figure 4:**
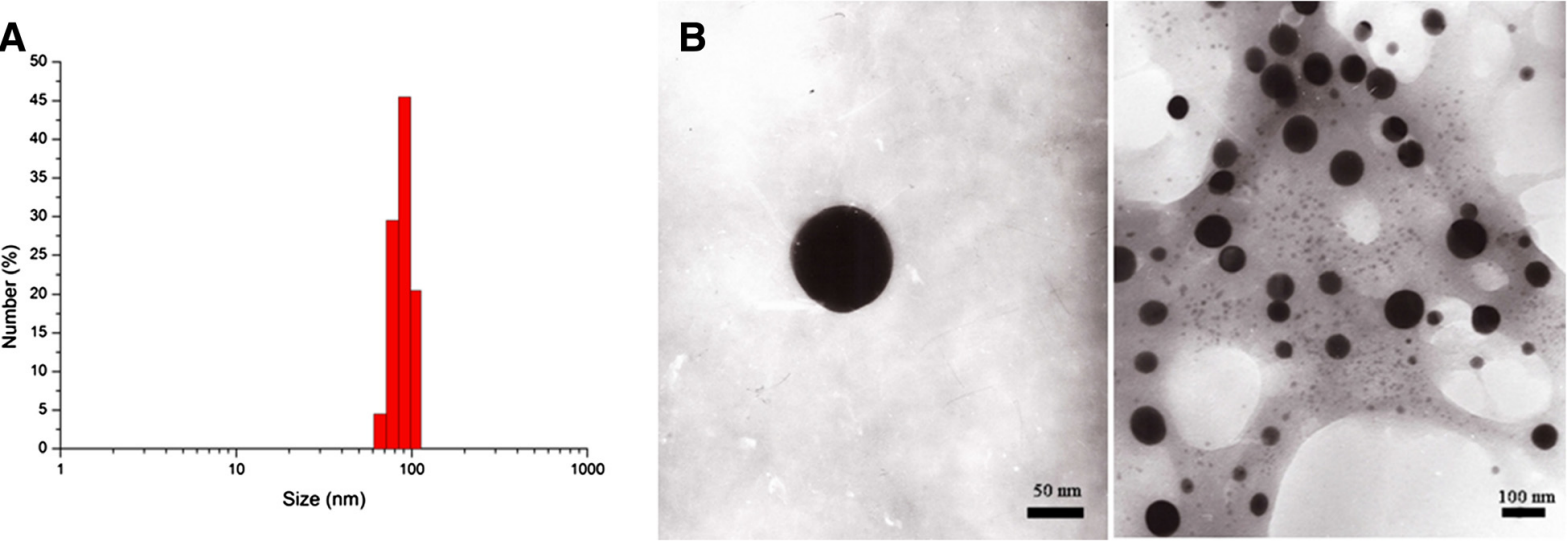
**Particle size distribution (A) and TEM image (B) of the obtained Gefitinib**-**loaded FA**-**BSA**-**CM**-**β**-**CD NPs.**

Using transmission electronic microscope (TEM), we observed that Gefitinib-loaded FA-BSA-CM-β-CD NPs we prepared were monodisperse spheres, and further analysis revealed that the diameters of NPs ranged from 52.1 to 105.6 nm (Figure 4). Table 1 summarized the average diameters measured by dynamic light scattering (DLS) and surface charge information of these prepared nanoparticles. It was remarkable that Gefitinib-loaded FA-BSA-CM-β-CD NPs showed smaller particle size, negative zeta potential. The average encapsulation efficiency of Gefitinib in FA-BSA-CM-β-CD NPs was 89.2% and about 70.1% of FA was conjugated on the surface of NPs.

**Table 1:** **Key parameters of Gefitinib**-**loaded FA**-**BSA**-**CM**-**β**-**CD NPs**

**Parameters**	**Data**
Average diameter (nm)	90.2 ± 10.01
Zeta potential (mv)	−18.6 ± 5.8
Encapsulating efficiency of Gefitinib (%)	89.2 ± 4.7
Entrapment efficiency of FA (%)	70.1 ± 4.3
Polydispersity Index (PDI)	0.093

### In vitro drug release study

**Figure 5:**
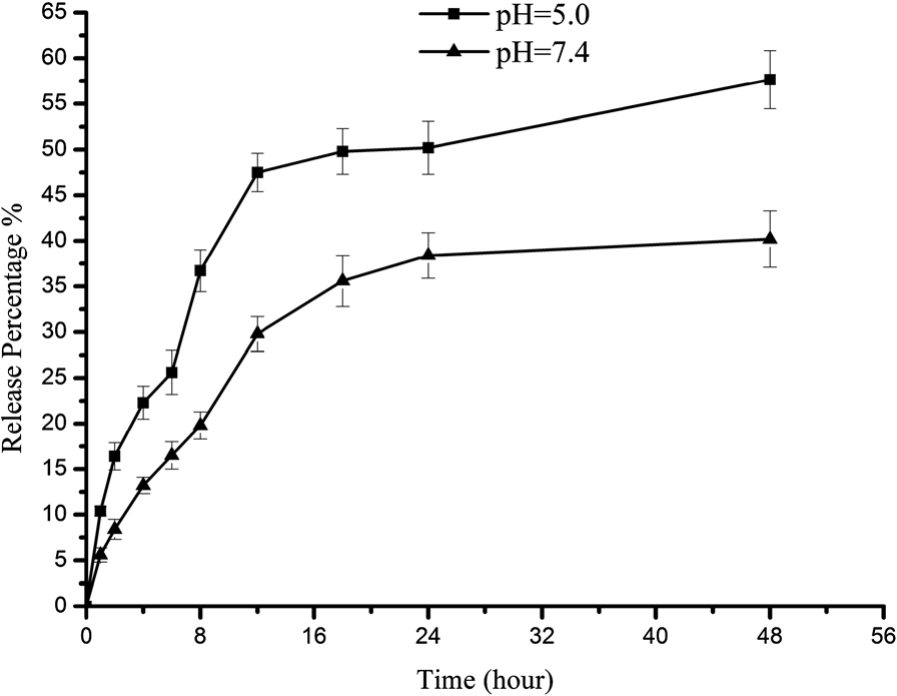
**Gefitinib release profiles from FA-BSA-CM-β-CD NPs in aqueous solution at 37°C.** Data were presented as mean ± SD (n = 3).

Gefitinib loaded FA-BSA-CM-β-CD NPs exhibited similar release profiles in the medium with different pH. The release curve in PBS could be divided into two phases: initial fast drug release stage and later stable release stage. Gefitinib was released rapidly in the initial fast release stage, and was released slowly in the later stable stage through diffusion because of the continuous degradation of the polymer (Figure 5). The release speed of Gefitinib decreased with the increase of pH as polymer degraded faster in acid medium. Thus, it possibly suggested that the matrix of NPs tended to be eroded as a result of depolymerization of BSA at pH 5.0 which was closer to the isoelectric points of BSA (pH 4.9), and then drug could be more easily released from NPs. The release ratio during the first 48 h accounted for over 40% of the total drug and the remnants were released over longer time of incubation. It suggested that Gefitinib-loaded FA-BSA-CM-β-CD NPs could be used as a long-lasting and effective drug delivery system.

### Cell viability assays

**Figure 6:**
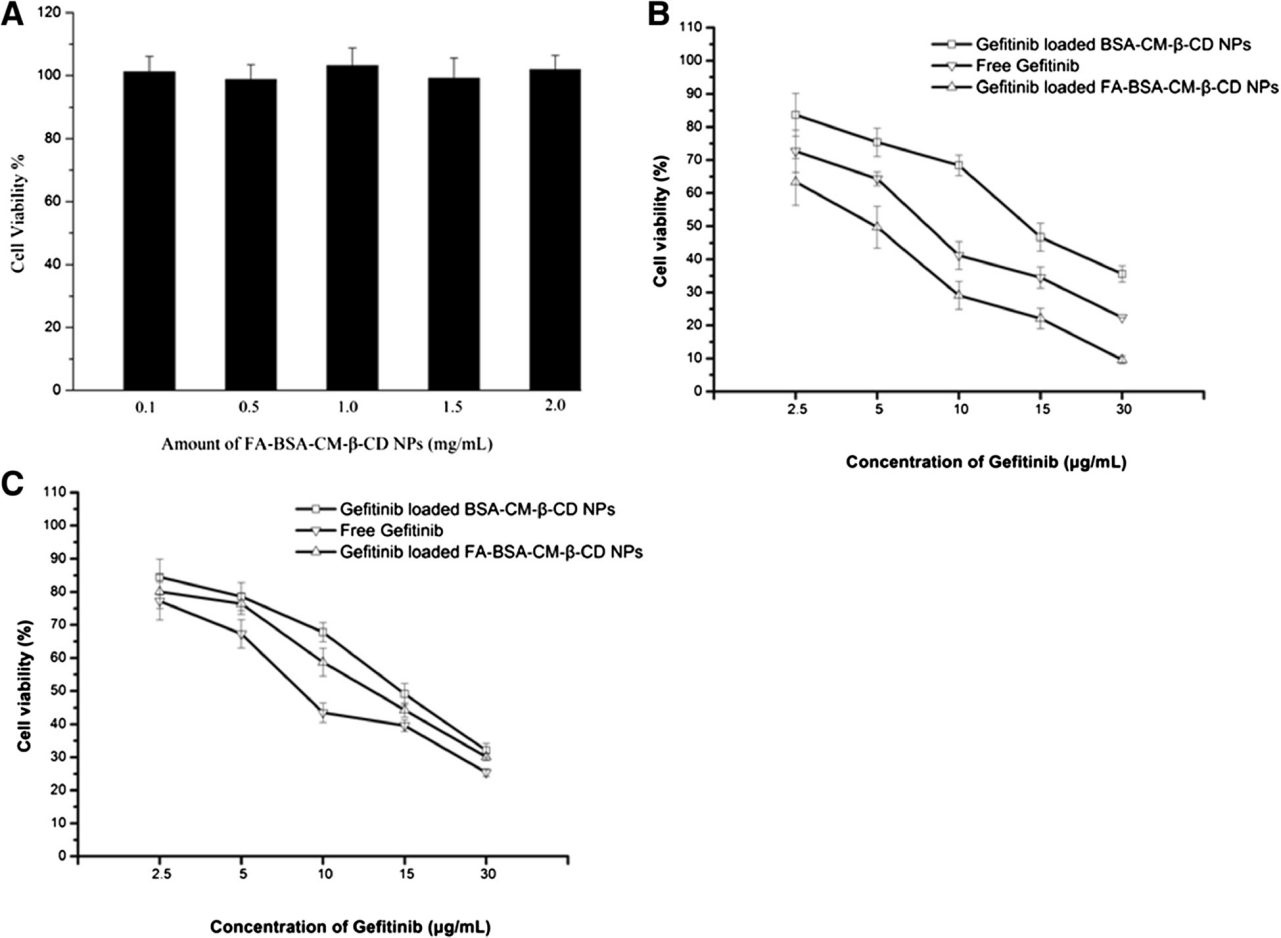
***In vitro*** **viability of NPs in Hela cells.** Data represents mean ± SD (n = 3). **(A)** Viability of Hela cells after incubation with different amounts of naked FA-BSA-CM-β-CD NPs after 48 h. **(B)** Cell viability cultured with NPs loaded at various concentrations of Gefitinib in folate-free medium after 48 h. **(C)** Cell viability cultured with Gefitinib loaded NPs at various concentrations in medium containing FA at 5 μg/mL after 48 h.

The cytotoxic effects of Gefitinib loaded FA-BSA-CM-β-CD NPs and BSA-CM-β-CD NPs were evaluated by MTT assay using Hela cell line. MTT analysis showed that in the absence of FA in culturing medium, treatment of Hela cells with Gefitinib loaded FA-BSA-CM-β-CD NPs caused a markedly increase in the cell cytotoxic activities as compared with free Gefitinib and Gefitinib loaded FA unconjugated NPs (Figure 6B). The IC_50_ values of Gefitinib loaded FA-BSA-CM-β-CD NPs treated Hela cells was 4.63 μg/mL, 7.85 μg/mL for free Gefitinib and 13.55 μg/mL for Gefitinib loaded BSA-CM-β-CD NPs. However, no obvious cytotoxic activities were observed when treating the cells with blank FA-BSA-CM-β-CD NPs in Hela cells (Figure 6A). These data suggested that more drug loaded FA conjugated NPs could be internalized into Hela cells which expressed FA at higher level by the interaction between FA and FR, further leading to the significant cytotoxicity by the accumulation of drug in cells.

We next examined whether inhibition of FR, which expressed on the cell surface, affected the cytotoxic activities of Gefitinib loaded FA-BSA-CM-β-CD NPs. Hela cells were cultured in the medium containing FA at 5 μg/mL. MTT analysis (Figure 6C) revealed that the presence of FA caused a significant decrease in the cytotoxic effect of Gefitinib loaded FA-BSA-CM-β-CD NPs compared with that without FA in Hela cells. However, pretreatment of FA had little effect on the cytotoxic activity of free Gefitinib and Gefitinib loaded BSA-CM-β-CD NPs. The IC_50_ values of Gefitinib loaded FA-BSA-CM-β-CD NPs treated Hela cells was 13.02 μg/mL, 8.63 μg/mL for free Gefitinib and 14.76 μg/mL for Gefitinib loaded BSA-CM-β-CD NPs. These data further demonstrated that FA conjugation played critical roles in accumulating NPs inside FR-positive tumor cells and could be used as a targeting molecule in the treatment of human cancers which over-expressed FR on the cell surface.

### *In vitro* uptake ability analysis

**Figure 7:**
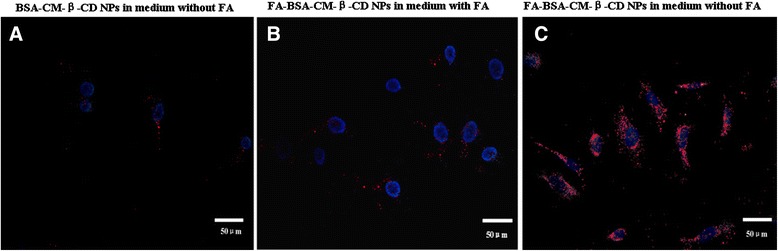
***In vitro*** **uptake ability for NPs.** Fluorescent image of the uptake of BSA-CM-β-CD NPs in medium without FA **(A)**. Fluorescence image of the uptake of FA-BSA-CM-β-CD NPs in medium with FA **(B)** and without FA **(C)**.

To visualize whether FA conjugation could enhance the uptake of BSA-CM-β-CD NPs, The NPs were labeled with Rodamine B and the uptake ability was evaluated in Hela cells. Using confocal laser scanning microscopy analysis, Hela cells showed increased number of red fluorescence patches in the cytoplasm when incubating Rhodamine B-labeled FA-BSA-CM-β-CD NPs in the FA-free medium for 6 h compared with that of Rhodamine B-labeled BSA-CM-β-CD NPs. However, the uptake of FA-BSA-CM-β-CD NPs was significantly reduced by addition of FA in the medium (Figure 7). The free FA competition study suggested that free FA in medium competed to bind FR on the surface of Hela cells with FA conjugated NPs, leading to the lower uptake of NPs.

### Intracellular ATP level assay

**Figure 8:**
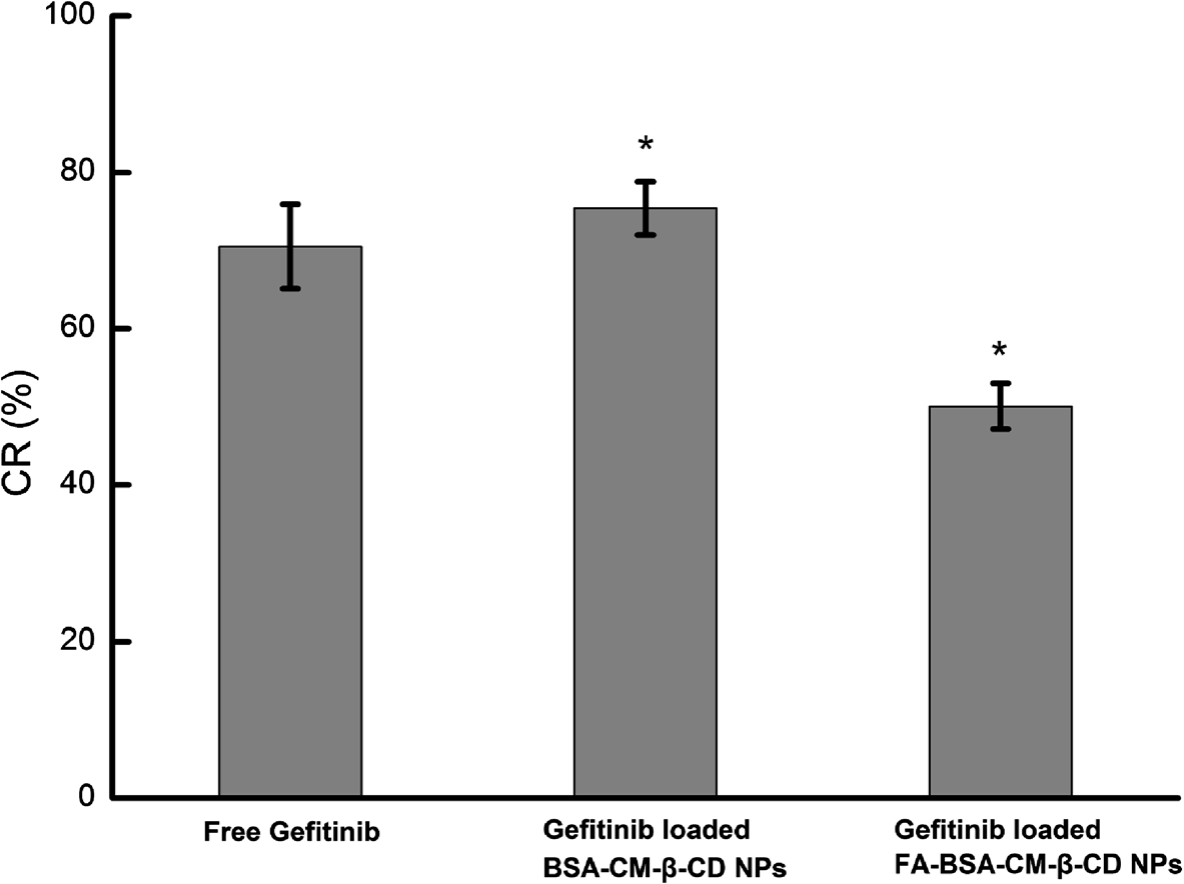
**Results of intracellular ATP level assay after 48 h incubation with different preparations.** Data were expressed as mean ± SD (n = 3). *P <0.05, vs the free Gefitinib group.

After cells were treated with free Gefitinib, Gefitinib loaded BSA-CM-β-CD NPs, Gefitinib loaded FA-BSA-CM-β-CD NPs, the changing rates of intracellular ATP level were presented in Figure 8. It can be seen that compared with ATP level of untreated Hela cells as the control group, The changing rates of intracellular ATP level for free Gefitinib, Gefitinib loaded BSA-CM-β-CD NPs and Gefitinib loaded FA-BSA-CM-β-CD NPs were decreased to 70.5%, 75.4% and 50.1%, respectively. The results showed that Gefitinib and Gefitinib loaded NPs were internalized to induce the apoptosis of cells by lowering ATP level rates. It also confirmed that with the interaction between FA conjugated on the surface of NPs and FR situated at Hela cells, more drug loaded FA conjugated NPs were transported into the interior of cells to inhibit energy generation and accelerate the apoptosis of cells by accumulation of drugs in cells.

### Cell apoptosis analysis

**Figure 9:**
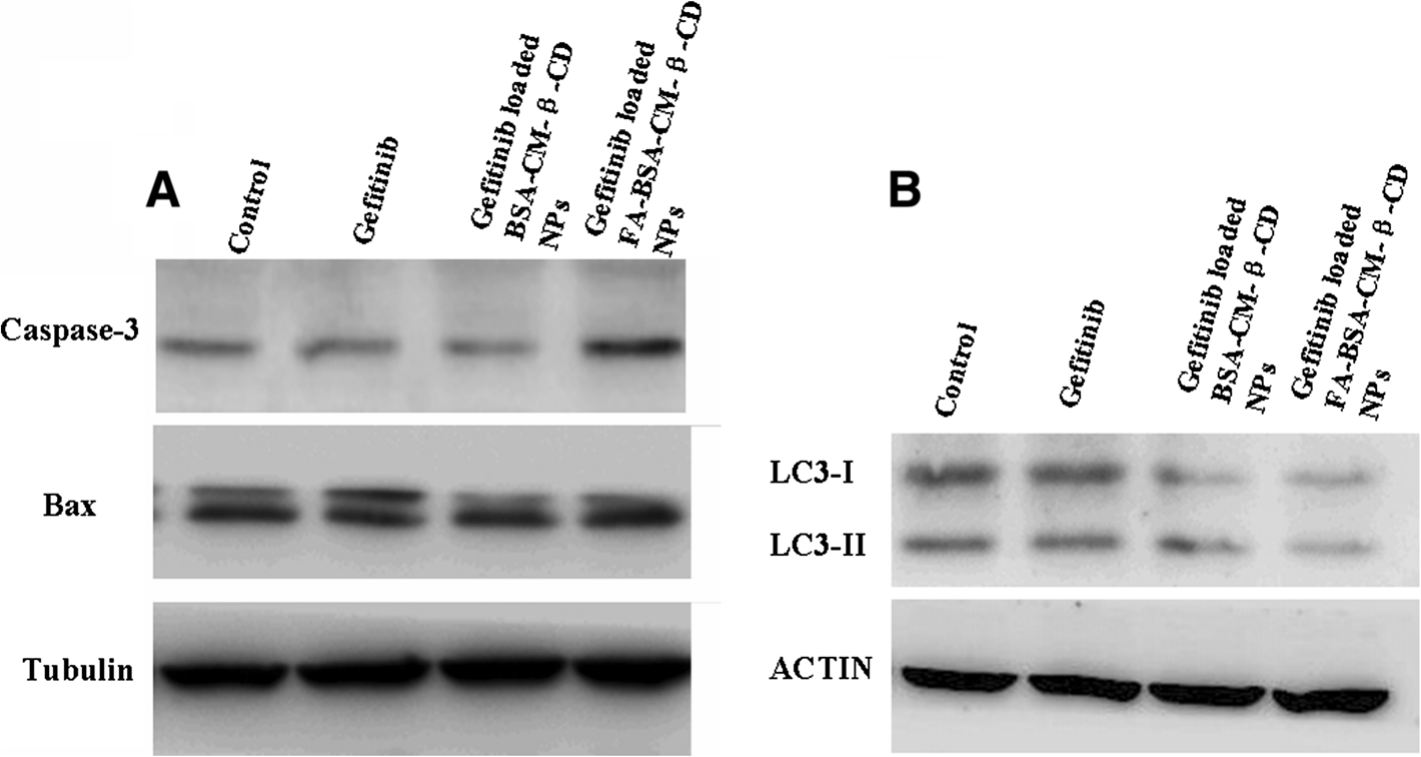
**Apoptotic effects of various Gefitinib formulations on Hela cells. (A)** Western blot analysis of the expression levels of Bax and caspase-3 proteins in Hela cells after treatments. **(B)** Western blot analysis of the expression levels of LC3 proteins in Hela cells after treatments.

To identify the effect of Gefitinib and Gefitinib loaded NPs on cell apoptosis and autophagy, we detected the expression of caspase-3, Bax, and LC3 by western blot (Figure 9). Compared with free Gefitinib and Gefitinib loaded NPs, Gefitinib loaded FA-BSA-CM-β-CD NPs induced the highest caspase-3 protein expression. It also illustrated that with the mediation of FA, a large amount of drug loaded FA conjugated NPs were accumulated in Hela cells and caspase-3 as the main apoptosis relevant protein was increased, corresponding with the results of MTT experiments. However, there was no obvious difference on the Bax protein expression in the treated groups and the control group, confirming that Bax was not involved in Gefitinib induced cell apoptosis (Figure 9A). LC3 (microtubule-associated protein light chain 3) is a specific autophagic marker in mammalian cells during autophagy. So, to identify whether Gefitinib affects autophagy, expression of LC3 was detected in Hela cells, and found that free Gefitinib did not influence the expression of LC3, but with the addition of NPs, the expression of LC3 has been inhibited, also, with the mediation of FA, the inhibition rate increased obviously (Figure 9B). So, the results suggested that through autophagy, Hela cells may be survival and resist free Gefitinib, and FA-NPs mediated accumulation of Gefitinib in cells inhibits LC3 expression. Taken together, through inhibition of autophagy, Gefitinib loaded FA-BSA-CM-β-CD NPs induced cells apoptosis.

### Inhibition of various endocytosis assay

**Figure 10:**
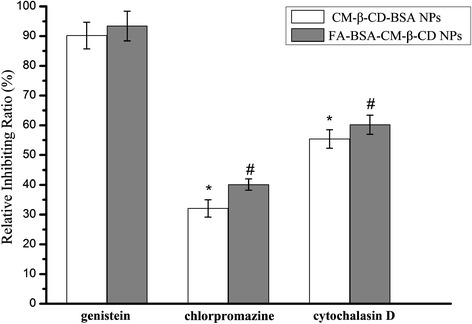
**Effects of endocytic inhibitors on the uptaking ability of the two NPs in Hela cells.** ^*^P <0.05, vs the BSA-CM-β-CD NPs group treated with genistein in Hela cells, ^#^P <0.05, vs the FA-BSA-CM-β-CD NPs group treated with genistein in Hela cells.

To get more insight to know which uptake mechanisms were implied in NPs uptake, Hela cells were pretreated with various endocytic inhibitors specific for a particular endocytic pathway. Figure 10 showed that when genistein as an inhibitor to block caveolae-mediated endocytosis (CvME) was added into cells, there was no significant difference in both NPs internalization suggesting a minor role of CvME. When cells were treated with cytochalasin D (30 μM, macropinocytosis), the uptake ability of both NPs were significantly decreased to 55.4% and 60.2%. It was also observed that internalization of both NPs in cells with chlorpromazine treatment (clathrin-mediated endocytosis) was significant lower than that in untreated cells. Moreover, 40.1% reduction in FA-BSA-CM-β-CD NPs was observed in comparison with 32.1% reduction of intracellular uptake of BSA-CM-β-CD NPs. Some previous study have reported that different conjugation with targeting ligands, such as iRGD, siRNA and disaccharide, could enhance uptake or change the endocytosis pathway of NPs resulting in improving cytotoxicity to cancer cells [38–41]. The results demonstrated that both NPs were internalized into cells mainly depending on clathrin-mediated endocytosis and macropinocytosis being proved by the significant uptake reduction of both NPs in treated cells with chlorpromazine and cytochalasin D. In contrast, the regulation of caveolae-mediated endocytosis on NPs internalization was not significantly different from untreated group.

## Conclusions

In summary, CM-β-CD was conjugated with BSA by carbodiimide coupling. FA as a small targeting molecule, was bond to the surface of NPs. Gefitinib loaded FA-BSA-CM-β-CD NPs showed good monodispersity, negative charge and homogenous particle size. The encapsulating efficiency of Gefitinib and the release pattern were investigated *in vitro*. MTT results showed that no obvious cytotoxity was observed when incubating naked FA-BSA-CM-β-CD NPs with Hela cells. The free folic acid competition study showed that the cell inhibition of FA conjugated NPs in FR positive cells was significantly enhanced when FA was removed from the medium. Gefitinib loaded FA-BSA-CM-β-CD NPs inhibited cells autophagy by down-regulating LC3, and promoted apoptosis of Hela cells through the prevention of ATP generation and elevating the expression of caspase-3 protein. It also confirmed that clathrin-mediated endocytosis and macropinocytosis played an important role on the internalization of both NPs. Taken together, FA-BSA-CM-β-CD NPs presented the promising candidates as a folate receptor-positive tumor-targeting carrier via folate mediation.

## Methods

### Materials

BSA was purchased from Sigma (USA), Carboxymethyl-β-Cyclodextrin Sodium Salt (CM-β-CD) was purchased from Zhiyuan Bio-Technology Co., Ltd (China), 1-ethyl-3-(3-dimethylaminopropyl) carbodiimide (EDAC) and folic acid were obtained from Sigma Chemicals (St Louis, US). Gefitinib was purchased from Eastbang Pharmaceutical Co., Ltd (China). All other chemicals were of reagent grade and were used as received.

### Synthesis of BSA-CM-β-CD conjugates

8.0 mg of CM-β-CD was dissolved in 8.0 mL PBS buffer solution (pH 5.8). Then, the solution was added into a centrifuge tube in the presence of 40.0 mg EDC and 16.0 mg NHS. After the tube was rotated constantly for 1 h, 8.0 mg BSA were added into the tube. Subsequently, the tube was rotated overnight. Finally, the BSA-CM-β-CD conjugates were collected by dialysis filter with a dialysis bag with 1000 molecular weight cutoff to remove the uncoupled CM-β-CD.

### Preparation of Gefitinib loaded FA-BSA-CM-β-CD self-assembled nanoparticles

20 mg conjugates were suspended in 20 mL of distilled water to achieve a solution with concentration of 1 mg/mL. Drug solution (0.4 mg/mL) was prepared by 2 mg Gefitinib was dissolved in 5 ml dichloromethane. Gefitinib loaded BSA-CM-β-CD NPs were prepared by quickly dropping 5.0 mL of drug solution into 20 mL distilled water containing 20 mg conjugates at 30°C under continuously stirring for 24 h to remove dichloromethane. FA was conjugated with BSA-CM-β-CD NPs as the following: 5 mg folic acid was dissolved in 10 mL phosphate buffer suspension (pH 7.4), then 15.0 mg EDC and 5.0 mg NHS were added under stirring constantly for 1 h. Entrapment efficiency of FA conjugated with BSA-CM-β-CD NPs was determined as the ratio of actual FA loading amount to the initial added FA amount. The solution was centrifuged at 16,000 rpm for 30 min. NPs collected were washed 3–4 times with deionized water and centrifuged at 16,000 rpm for 20 min, freeze drying to obtain powders.

### Characterization of Gefitinib loaded FA-BSA-CM-β-CD NPs

The characterization of Gefitinib loaded FA-BSA-CM-β-CD NPs was investigated by using AFFINITY-1 IR spectroscopy (Shimadzu, Kyoto, Japan). Its morphology was observed by using transmission electron microscope (TEM) (JEM-1200EX, Tokyo, Japan) and the mean diameter and zeta potential were determined by Zetasizer (Nano ZS90, Malvern, UK). The encapsulation efficiency (EE) of Gefitinib in FA-BSA-CM-β-CD NPs was calculated using the equation listed below.

$$ \mathrm{E}\mathrm{E}\left(\%\right) = \frac{\mathrm{Weight}\ \mathrm{of}\ \mathrm{in}\mathrm{itially}\ \mathrm{added}\ \mathrm{drug}\hbox{-} \mathrm{Weight}\ \mathrm{of}\kern0.5em \mathrm{free}\ \mathrm{drug}\ \mathrm{in}\ \mathrm{supernatant}}{\mathrm{Weight}\ \mathrm{of}\ \mathrm{in}\mathrm{itially}\ \mathrm{added}\ \mathrm{drug}}\times 100 $$

### Assessment of drug release

The drug releases were carried out in PBS containing 10% serum with different pH at 37.0 ± 0.5°C under gentle agitation. 10 mL PBS (pH 7.4) in which accurate weighed 10 mg dried Gefitinib-loaded FA-BSA-CM-β-CD NPs were suspended was put into a dialysis bag with 1000 molecular weight cutoff and the dialysis bag was immersed into 100 mL phosphate buffer solution containing 10% serum maintained at pH 7.4 or 5.0 at 37.0 ± 0.5°C. At predetermined intervals, 5 mL of release medium was withdrawn and the same volume of fresh buffer solution was added. Samples were filtered through 0.45 μm filter and the concentrations of Gefitinib released were analyzed by spectrophotometry at 338 nm.

### Cell viability assays

A 3-(4,5-dimethylthiazol-2-yl)-2,5- diphenyltetrazolium bromide (MTT) assay was used to investigate cell viability. Hela cells were chosen and used as a model folate receptor-positive cell line in angiogenesis targeting delivery and treatment for their high FR expression [42,43]. Hela cells were seeded into the 96-well plate at a density of 5 × 10^4^/mL and attached for 24 h at 37°C in both folate-free medium and the medium containing folate at 5 μg/mL under 5% CO_2_. Then, the cells were treated with free Gefitinib and Gefitinib-loaded NPs for 48 h, followed by addition of 20 μL MTT (5 mg/mL) and incubated for 4 h at 37°C. Then, the supernatant was carefully removed and 150 μL DMSO was added to each well and stirred for 30 min. The absorbance was measured using microplate reader at 490 nm.

### Uptaking ability of different kinds of NPs in Hela cells

Hela cells, (a folate receptor-positive cell line) a model cell line, were applied to investigate cell uptake ability of different kinds of nanoparticles. Rhodamine B as the fluorescent marker was encapsulated in NPs. Cells were cultured in flasks supplemented with both folate-free medium and the medium containing folate 5 μg/mL at 37°C and 5% CO_2_. When the cell concentrations reached 5 × 10^4^/mL, 100 μL of medium containing cells was transferred to the 96-well plate and treated with different Rhodamine B-labeled nanoparticles. At specified time, NPs were withdrawn and the wells were washed with PBS. Nucleus was stained with Hoechst for 15 min at 37°C followed by double washing with PBS. The internalization of RhB-loaded NPs into cells was observed using fluorescent microscopy (DMI400B, Leica, Germany).

### Intracellular ATP level assay

Free Gefitinib, Gefitinib loaded BSA-CM-β-CD NPs, Gefitinib loaded FA- BSA-CM-β-CD NPs at the same drug concentration were added into the 96-well plate filled with Hela cells at a density of 5 × 10^4^/mL for 48 h. The luciferin-luciferase-based ATP luminescence assay kit was applied to determine the changing rates of intracellular ATP level (CR%) calculated using equation below.

$$ \mathrm{C}\mathrm{R}\left(\%\right) = \frac{\mathrm{ATP}\ \mathrm{level}\ \mathrm{of}\ \mathrm{Hela}\ \mathrm{cells}\ \mathrm{treated}\ \mathrm{with}\ \mathrm{free}\ \mathrm{drug}\ \mathrm{or}\ \mathrm{N}\mathrm{P}\mathrm{s}}{\mathrm{ATP}\ \mathrm{level}\ \mathrm{of}\ \mathrm{untreated}\ \mathrm{Hela}\ \mathrm{cells}}\times 100 $$

### Tracking of uptake pathways using various endocytic inhibitors

In order to analyze the potential mechanism on uptake pathways of nanoparticles, three types of endocytic inhibitors including cytochalasin D (30 μM, macropinocytosis), genistein (1 μg/mL, caveolae mediated endocytosis) and chlorpromazine (10 μg/mL, clathrin mediated endocytosis) were preincubated with Hela cells in 96-well plate for 30 min, respectively. Then both FITC labeled BSA-CM-β-CD NPs and FA- BSA-CM-β-CD NPs were treated with cells to track the uptake pathways. The effects of various inhibitors on the uptake pathway of the NPs were evaluated by comparing the intracellular fluorescent intensity between treatment of adding inhibitors and non-inhibitors.

### Western blot assay

After treated with free Gefitinib, Gefitinib loaded BSA-CM-β-CD NPs, Gefitinib loaded FA-BSA-CM-β-CD NPs, cells were harvested, washed twice with ice cold PBS, then lysed in RIPA buffer (150 mM NaCl, 1% NP-40, 1% SDS, 1 mM PMSF, 10 μg/mL leupeptin, 1 mM aprotinin, 50 mM Tris-Cl, pH 7.4). The cell lysate was cleared by centrifugation at 12,000 × g for 25 min. Cell lysate containing 50 μg protein in 20 μL was separated by 10% SDS-PAGE and the protein was transferred onto polyvinylidene fluoride (PVDF) membrane. After blocking with 1% BSA, the PVDF membrane was incubated with the primary antibodies (caspase-3, Bax, tubulin, LC3) at 4°C overnight. Subsequently, incubated with appropriate secondary antibody for 1 h and stained with ECL. The level of the targeted proteins were photographed and analyzed by UVP gel analysis system.

## Abbreviations

FA: Folic acid
FR: Folate receptor
BSA: Bovine Serum Albumin
CM-β-CD: Carboxymethyl-β-Cyclodextrin
NPs: Nanoparticles
